# How to combat emerging artemisinin resistance: Lessons from “The Three Little Pigs”

**DOI:** 10.1371/journal.ppat.1006923

**Published:** 2018-04-26

**Authors:** Thanat Chookajorn

**Affiliations:** Genomics and Evolutionary Medicine Unit (GEM), Center of Excellence in Malaria Research, Faculty of Tropical Medicine, Mahidol University, Bangkok, Thailand; Washington University School of Medicine, UNITED STATES

## Abstract

It is rare to come across an Aesop’s fable in respectable journals. It might catch scientists outside the malaria field by surprise to learn that the famous story of “The Boy Who Cried Wolf” has been repeatedly compared to the threat from artemisinin-resistant malaria parasites, including the two latest reports on the rise of a specific haplotype in Cambodia and Thailand, sensationally dubbed “Super Malaria” by the media [1, 2]. The comparison to a children’s tale should not negate the fact that malaria drug resistance is one of the most pressing threats to the global public health community. Here, the findings leading to this contentious discourse will be delineated in order to provide a perspective. Possible solutions will be presented to stimulate further research and discussion to solve one of the greatest public health challenges of our lifetime.

Wolf: Little pig, little pig, let me in, let me in.

Pig: Not by the hair on my chinny chin chin!

Wolf: Then I’ll huff and I’ll puff and I’ll blow your house in.

—The Story of the Three Little Pigs.

## Living in the wolf’s lair: Should the world be worried about the threat of artemisinin resistance emerging in Southeast Asia?

To fully understand the controversy, one must look back at the history of malaria drug resistance. In the last century alone, we saw the rise and fall of several antimalarial compounds [3, 4]. Malaria parasites infect approximately 200 million people worldwide every year, and the only tool for treatment is a small arsenal of antimalarial drugs [5]. The pivotal losses of two key drugs (chloroquine and pyrimethamine) during the last few decades have led to millions of deaths [6]. Parasites from Southeast Asia acquired resistant mutations, rendering chloroquine and pyrimethamine useless, and these alleles have spread to Africa, causing a devastating increase in malaria mortality, especially from multidrug-resistant *Plasmodium falciparum* [7, 8]. The resistant alleles originate from parasite populations at the border between Thailand and Cambodia, the epicenter of malaria drug resistance, where parasites are prone to develop drug resistance [9]. Cultured parasite lines from Southeast Asia are often used as strains of choice for long-term in vitro drug-selection experiments due to their adaptability to drug pressure [10]. Even though the underlying mechanism is not clear, their genetic makeups allow them to evolve compensatory mechanism(s) to offset the loss of fitness incurred by deleterious drug-resistant mutations, as demonstrated in the situation of pyrimethamine resistance [11–13].

After the loss of chloroquine and pyrimethamine, what is at stake now is a new “miracle” drug in the form of artemisinin. Its discovery began during the Vietnam War by a Chinese research team commissioned to find new antimalarial regimens [14]. The fast parasite clearance action of artemisinin is unparalleled since it is the only drug that can kill every asexual red blood cell stage [14]. Implementation of artemisinin has turned the tide in the fight against malaria, resulting in a significant drop in malaria mortality [15]. Based on the history and the known origin of malaria drug resistance, Cambodia has become a sentinel site for monitoring emerging artemisinin resistance [16, 17]. There is now a large body of data tracking the time taken to clear parasites from malaria patients treated with artemisinin. A trend showing delayed clearance time by hours has become more pronounced in recent years [17, 18]. It is important to note that the drug can still kill parasites, but it takes longer to achieve clearance. This change in artemisinin susceptibility does not fit a conventional definition of drug resistance because treatment failure by the recommended drug regimen and dosage has not yet been observed. The term “resistance” would fit in the sense that the number of parasites killed by artemisinin is reduced. Even though parasites with delayed clearance time following artemisinin treatment have now been detected throughout Cambodia and parts of Thailand, the use of the term “artemisinin resistance” to describe them is understandably controversial.

The malaria research community has sought potential molecular markers to monitor the artemisinin resistance situation. The use of such markers in simple and rapid molecular tests would widen the scope of resistance monitoring, which is currently limited to sites where elaborate clinical observation can be conducted. Genetic changes at the *kelch 13* gene have been recognized by several research laboratories as a marker for reduced artemisinin susceptibility [19, 20]. Certain *kelch 13* alleles are associated with the decline in clinical efficiency of the artemisinin-based regimen [21]. The association between *kelch 13* mutant alleles and reduced artemisinin susceptibility has been proven experimentally by an in vitro laboratory assay in combination with transgenic parasite lines [22]. However, the study of emerging artemisinin resistance is not straightforward, since the increase in the half-maximal inhibitory concentration (IC_50_), a standard measurement of drug sensitivity, is small [23]. A robust drug resistance could increase the IC_50_ level by at least 2 orders of magnitude, but the shift in artemisinin sensitivity from field isolates obtained from patients with delayed clearance is minuscule (2- to 3-fold) [23]. This is consistent with the absence of any report of complete treatment failure, but makes any association study experimentally difficult. An alternative method for measuring artemisinin sensitivity relies on determining the number of surviving parasites after exposure to a short pulse of artemisinin [24]. This kind of survival assay was shown to be correlated with delayed clearance and was implemented to show a functional association between reduced artemisinin susceptibility and certain *kelch 13* mutations [22]. The survival assay and molecular tests for *kelch 13* mutations are being used as the tools to herald the crisis of artemisinin resistance with the same ardency as chloroquine and pyrimethamine resistance. Nevertheless, for clinicians in the field, artemisinin-based combination therapies are still the drug regimen of choice for treating *P*. *falciparum* malaria, even in the areas where parasites show reduced artemisinin susceptibility. A sensational warning of the looming global threat from artemisinin resistance was perceived as a scaremongering tactic by many others, leading to the comparison of artemisinin resistance to the fable of “The Boy Who Cried Wolf” [6, 25–28].

When all the clarion calls are damped down, the emergence of *P*. *falciparum* parasites demonstrating reduced artemisinin susceptibility (albeit small at present) is an incontrovertible (even if inconvenient) fact. The well-known moral lesson of “The Boy Who Cried Wolf” is for teaching children not to misrepresent the truth. However, the wolf did eventually show up when everyone ignored the threat. In this case, the wolf is full-blown artemisinin resistance capable of surviving existing artemisinin treatment regimens. It is undeniable that more and more parasites in Southeast Asia have become less sensitive to artemisinin [18]. The rise in recrudescence (return of malaria parasites after treatment within 28–42 days) has become noticeable, which could be the result of either incomplete clearance by artemisinin or the failure of partner drugs—or perhaps both [29]. Another troubling fact is that the loss of artemisinin susceptibility has emerged at the epicenter of malaria multidrug resistance. If malaria drug resistance is compared to a wolf, the area at the border between Cambodia and Thailand could be considered a wolf’s lair packed with the parasites conferring resistance to almost every clinically implemented antimalarial drug. Parasite populations in this location have a strong propensity to develop resistance, and complete artemisinin resistance will be a deadly addition to their ability to withstand several antimalarial drug regimens. With all things considered, the question is not whether the wolf exists, but how big of a threat it poses.

## What is the proportional response to a lurking wolf? Here come three little pigs

The year 2017 was an eventful one in malaria drug resistance research, with two key new findings related to a partner drug of artemisinin. In general, artemisinin is not administered alone since it has a short half-life, leaving remnant surviving parasites to propagate and develop resistance [14]. Artemisinin derivatives are paired with matching antimalarial partners, and the two widely used regimens in Southeast Asia are artesunate-mefloquine and dihydroartemisinin-piperaquine [30]. In Cambodia, parasites were found to return after dihydroartemisinin-piperaquine treatment [31, 32]. Two reports identified copy number polymorphism of a gene encoding one of the hemoglobin protease enzymes as a genetic marker for piperaquine resistance [33, 34]. Since these findings were published, it has been observed that parasites containing a unique combination of the molecular marker alleles for artemisinin and piperaquine resistance have spread at the border areas between Cambodia and Thailand [2, 35]. The parasite population with the combination of a C580Y *kelch 13* mutation and a hemoglobin protease copy number polymorphism has predominated Cambodia at an alarming rate. This is portrayed as a catastrophic event with a dangerous strain sweeping through the provinces on both sides of the Thai–Cambodian border. Nevertheless, there is no indication that artemisinin resistance has reached the level of treatment failure. The World Health Organization issued a statement to assure the public that the health community can still treat malaria patients with the currently available drugs [36]. Heated exchanges among malaria researchers have provoked a renewed comparison to “The Boy Who Cried Wolf” [26].

A better and perhaps more productive analogy is that of “The Three Little Pigs.” The three piglets in the story built their homes using different materials. Each piglet strategizes its investment in the choice of building material. When the wolf attacks, the houses built from straw and wood are annihilated. Only the brick house could withstand the attack. With the benefit of hindsight, the choice of building a brick house is appropriate for combating the wolf, but the investment is greater than that of the other two types of material. The same thing could be said about withstanding the threat from emerging parasites with reduced artemisinin sensitivity. If the level of resistance is not increased, the current regimens with artemisinin derivatives will be sufficient to control the malaria situation in Southeast Asia. It would not require a large investment, comparable to building a house from straw. However, there is no guarantee that the situation will not get worse, and it might now be the time to build a better and sturdier house.

**Fig 1:**
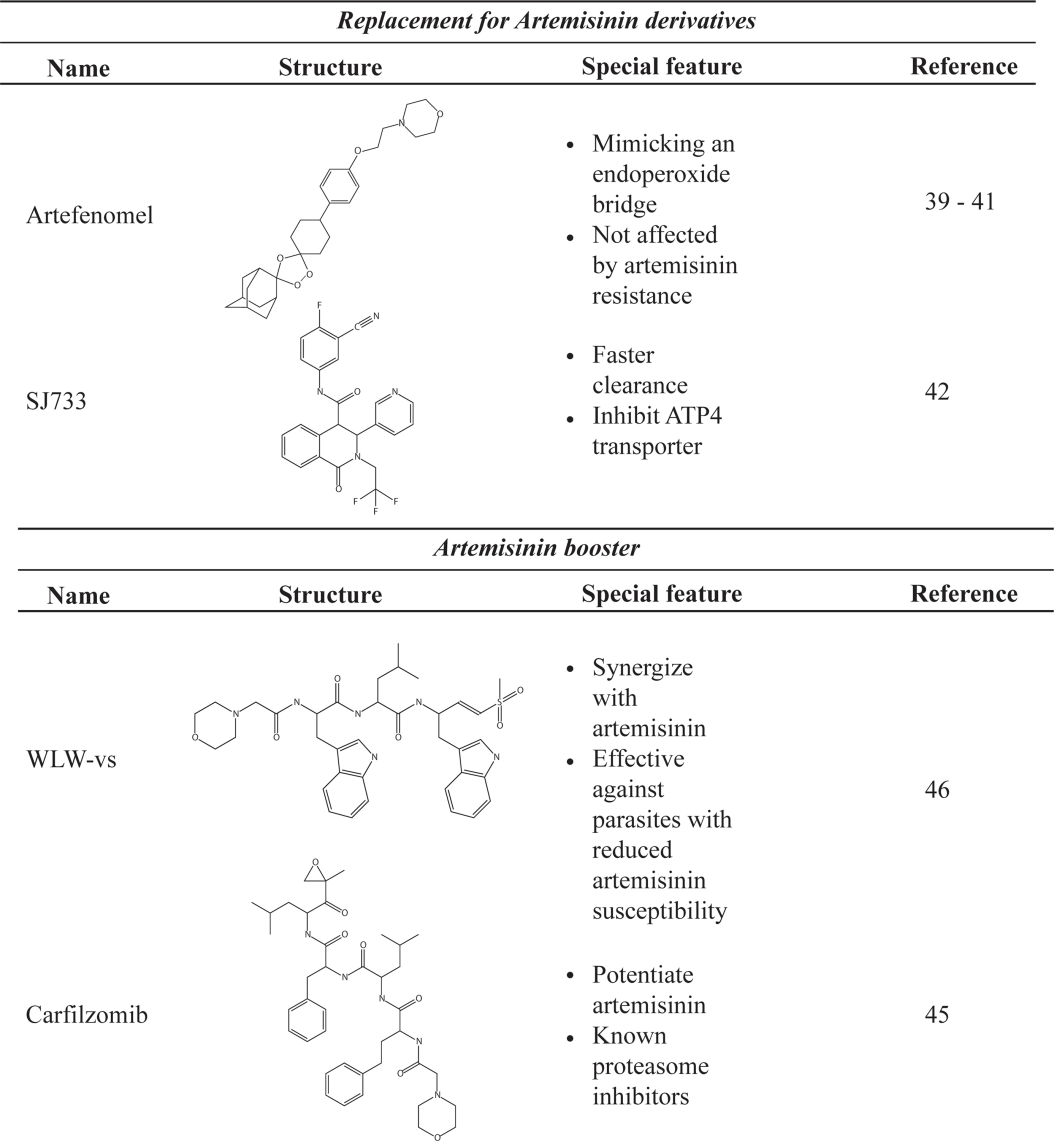
List of compounds that can be used against artemisinin-resistant parasites.

Malaria drug discovery, though requiring considerably more time and resources, could be a solution to emerging artemisinin resistance. The simplest and most straightforward solution is to introduce a third clinically available drug into the existing artemisinin combination therapy. Clinical trials of triple combinations, dihydroartemisinin + piperaquine + mefloquine and artemether + lumefantrine + amodiaquine, are ongoing to study their efficacy and to determine how well malaria patients could tolerate the triple regimen [37, 38]. If a safe triple regimen is developed, it should slow down the emergence of artemisinin-resistant parasites. However, this is likely to be a short-term solution. The ideal solution, equivalent to a brick house, is to develop novel compounds that directly combat the threat of emerging artemisinin resistance. The drug candidates for combating artemisinin resistance could be either (i) a replacement for artemisinin or (ii) an artemisinin activity booster (Fig 1).

In case (i), a compound must be able to target every pathogenic red blood cell stage. To mimic the success of artemisinin, a collection of compounds with an endoperoxide bridge—a chemical moiety responsible for the antimalarial activity of artemisinin—was explored, resulting in the discovery of the antimalarial activity of 1,2,4-trioxolane [39]. At present, artefenomel (OZ439), a trioxolane compound with improved pharmacokinetic properties, is in Phase IIb of the drug development pipeline [40]. A shift in artefenomel sensitivity was not observed in the parasites with reduced artemisinin susceptibility [41]. Another excellent alternative is SJ733. It is fast-acting, even more effective than artemisinin in parasite reduction, and is capable of killing ring-stage parasites, a property shared only with artemisinin among current drugs [42]. Moreover, SJ733 targets a parasite transporter protein with a novel mode of action distinct from other drugs [42]. Although still in the early stage of clinical development, SJ733 has shown great potential. These two new drug candidates are presented as potential replacements for artemisinin because of their ability to quickly clear the parasite load. This property is essential in the treatment of severe malaria patients whose bodies are flooded with infected red blood cells at the time of hospitalization.

Another important, but largely unexplored, property is that of compounds that can enhance the killing activity of artemisinin, especially against parasites with reduced susceptibility to artemisinin. At present, the mechanism underlying decreased artemisinin susceptibility is not fully understood, but factors with functions in protein homeostasis are genetically associated with the shift in artemisinin sensitivity [43, 44]. Hence, known proteasome inhibitors were tested against parasites with reduced artemisinin susceptibility and found to improve the killing activity of artemisinin [45]. To improve compound specificity and reduce host toxicity, analogues of peptides specifically targeted by the *P*. *falciparum* proteasome were developed [46]. These analogues can synergize with artemisinin and partially improve its activity [46]. These artemisinin booster compounds are still in the early stage of development, but they have already shown great potential. Since artemisinin is a precious life-saving drug, developing a booster compound might be a better strategy than finding a replacement.

## Concluding remarks

Currently, the threat from artemisinin resistance is growing as parasites containing specific resistant alleles are spreading. It is not possible to predict whether the reduction in the level of drug sensitivity will rise to the level of artemisinin treatment failure. The absence of full-blown artemisinin resistance is in line with the hypothesis that a highly reactive heme-activated artemisinin targets multiple proteins, as shown in two independent proteomic studies [47, 48]. The gain of mutations affecting a particular target of artemisinin alone is unlikely to completely abrogate the antimalarial activity of artemisinin. Nevertheless, the malaria research community once mistook the efficacy of chloroquine for ultimate success in the fight against malaria [49]. When chloroquine resistance appeared, the damage to malaria-control efforts was devastating [8]. Possible strategies to contain the problem of reduced artemisinin susceptibility range from testing new drug regimens consisting of multiple combinations of existing antimalarial drugs to an ambitious elimination program to get rid of malaria parasites in Southeast Asia. A drug discovery project to either find compounds that can work as effectively as artemisinin or even to improve the antimalarial activity of artemisinin will be a large investment in time and resources. However, until the nature of the threat is fully appreciated, having the strongest brick house could save lives in the long run.

## References

[1] Amato R, Pearson RD, Almagro-Garcia J, Amaratunga C, Lim P, Suon S, et al Origins of the current outbreak of multidrug-resistant malaria in southeast Asia: a retrospective genetic study. The Lancet Infectious Diseases. doi: 10.1016/S1473-3099(18)30068-9 29398391 10.1016/S1473-3099(18)30068-9 PMC5835763

[2] Imwong M, Hien TT, Thuy-Nhien NT, Dondorp AM, White NJ. Spread of a single multidrug resistant malaria parasite lineage (PfPailin) to Vietnam. Lancet Infect Dis. 2017;17(10):1022–3. Epub 2017/09/28. doi: 10.1016/S1473-3099(17)30524-8 . 28948924 10.1016/S1473-3099(17)30524-8

[3] Packard RM. The origins of antimalarial-drug resistance. The New England journal of medicine. 2014;371(5):397–9. Epub 2014/07/31. doi: 10.1056/NEJMp1403340 . 25075832 10.1056/NEJMp1403340

[4] D'Alessandro U, Buttiens H. History and importance of antimalarial drug resistance. Trop Med Int Health. 2001;6(11):845–8. Epub 2001/11/13. . 11703837 10.1046/j.1365-3156.2001.00819.x

[5] White NJ, Pukrittayakamee S, Hien TT, Faiz MA, Mokuolu OA, Dondorp AM. Malaria. Lancet. 2014;383(9918):723–35. doi: 10.1016/S0140-6736(13)60024-0 . 23953767 10.1016/S0140-6736(13)60024-0

[6] White NJ. Counter perspective: artemisinin resistance: facts, fears, and fables. Am J Trop Med Hyg. 2012;87(5):785 doi: 10.4269/ajtmh.2012.12-0573 . 23136172 10.4269/ajtmh.2012.12-0573 PMC3516251

[7] Roper C, Pearce R, Nair S, Sharp B, Nosten F, Anderson T. Intercontinental spread of pyrimethamine-resistant malaria. Science. 2004;305(5687):1124 doi: 10.1126/science.1098876 . 15326348 10.1126/science.1098876

[8] Wellems TE, Plowe CV. Chloroquine-resistant malaria. J Infect Dis. 2001;184(6):770–6. doi: 10.1086/322858 . 11517439 10.1086/322858

[9] Rathod PK, McErlean T, Lee PC. Variations in frequencies of drug resistance in Plasmodium falciparum. Proc Natl Acad Sci U S A. 1997;94(17):9389–93. . 9256492 10.1073/pnas.94.17.9389 PMC23200

[10] Flannery EL, Fidock DA, Winzeler EA. Using genetic methods to define the targets of compounds with antimalarial activity. J Med Chem. 2013;56(20):7761–71. doi: 10.1021/jm400325j ; PubMed Central PMCID: PMC3880619. 23927658 10.1021/jm400325j PMC3880619

[11] Kumpornsin K, Kotanan N, Chobson P, Kochakarn T, Jirawatcharadech P, Jaru-ampornpan P, et al Biochemical and functional characterization of Plasmodium falciparum GTP cyclohydrolase I. Malar J. 2014;13:150 doi: 10.1186/1475-2875-13-150 ; PubMed Central PMCID: PMC4005822. 24745605 10.1186/1475-2875-13-150 PMC4005822

[12] Kumpornsin K, Modchang C, Heinberg A, Ekland EH, Jirawatcharadech P, Chobson P, et al Origin of robustness in generating drug-resistant malaria parasites. Mol Biol Evol. 2014;31(7):1649–60. doi: 10.1093/molbev/msu140 ; PubMed Central PMCID: PMC4069624. 24739308 10.1093/molbev/msu140 PMC4069624

[13] Heinberg A, Siu E, Stern C, Lawrence EA, Ferdig MT, Deitsch KW, et al Direct evidence for the adaptive role of copy number variation on antifolate susceptibility in Plasmodium falciparum. Mol Microbiol. 2013;88(4):702–12. doi: 10.1111/mmi.12162 ; PubMed Central PMCID: PMC3654098. 23347134 10.1111/mmi.12162 PMC3654098

[14] White NJ. Qinghaosu (artemisinin): the price of success. Science. 2008;320(5874):330–4. doi: 10.1126/science.1155165 . 18420924 10.1126/science.1155165

[15] Ndeffo Mbah ML, Parikh S, Galvani AP. Comparing the impact of artemisinin-based combination therapies on malaria transmission in sub-Saharan Africa. Am J Trop Med Hyg. 2015;92(3):555–60. Epub 2015/01/28. doi: 10.4269/ajtmh.14-0490 ; PubMed Central PMCID: PMCPMC4350548. 25624402 10.4269/ajtmh.14-0490 PMC4350548

[16] Denis MB, Tsuyuoka R, Poravuth Y, Narann TS, Seila S, Lim C, et al Surveillance of the efficacy of artesunate and mefloquine combination for the treatment of uncomplicated falciparum malaria in Cambodia. Trop Med Int Health. 2006;11(9):1360–6. Epub 2006/08/26. doi: 10.1111/j.1365-3156.2006.01690.x . 16930257 10.1111/j.1365-3156.2006.01690.x

[17] Dondorp AM, Nosten F, Yi P, Das D, Phyo AP, Tarning J, et al Artemisinin resistance in Plasmodium falciparum malaria. N Engl J Med. 2009;361(5):455–67. doi: 10.1056/NEJMoa0808859 . 19641202 10.1056/NEJMoa0808859 PMC3495232

[18] Ashley EA, Dhorda M, Fairhurst RM, Amaratunga C, Lim P, Suon S, et al Spread of artemisinin resistance in Plasmodium falciparum malaria. N Engl J Med. 2014;371(5):411–23. doi: 10.1056/NEJMoa1314981 ; PubMed Central PMCID: PMC4143591. 25075834 10.1056/NEJMoa1314981 PMC4143591

[19] Ariey F, Witkowski B, Amaratunga C, Beghain J, Langlois AC, Khim N, et al A molecular marker of artemisinin-resistant Plasmodium falciparum malaria. Nature. 2014;505(7481):50–5. doi: 10.1038/nature12876 . 24352242 10.1038/nature12876 PMC5007947

[20] White NJ. Malaria: a molecular marker of artemisinin resistance. Lancet. 2014;383(9927):1439–40. doi: 10.1016/S0140-6736(14)60656-5 . 24766952 10.1016/S0140-6736(14)60656-5

[21] Phyo AP, Ashley EA, Anderson TJC, Bozdech Z, Carrara VI, Sriprawat K, et al Declining Efficacy of Artemisinin Combination Therapy Against P. Falciparum Malaria on the Thai-Myanmar Border (2003–2013): The Role of Parasite Genetic Factors. Clin Infect Dis. 2016;63(6):784–91. Epub 2016/06/18. doi: 10.1093/cid/ciw388 ; PubMed Central PMCID: PMCPMC4996140. 27313266 10.1093/cid/ciw388 PMC4996140

[22] Straimer J, Gnadig NF, Witkowski B, Amaratunga C, Duru V, Ramadani AP, et al Drug resistance. K13-propeller mutations confer artemisinin resistance in Plasmodium falciparum clinical isolates. Science. 2015;347(6220):428–31. doi: 10.1126/science.1260867 . 25502314 10.1126/science.1260867 PMC4349400

[23] Chotivanich K, Tripura R, Das D, Yi P, Day NP, Pukrittayakamee S, et al Laboratory detection of artemisinin-resistant Plasmodium falciparum. Antimicrob Agents Chemother. 2014;58(6):3157–61. doi: 10.1128/AAC.01924-13 ; PubMed Central PMCID: PMC4068498. 24663013 10.1128/AAC.01924-13 PMC4068498

[24] Witkowski B, Amaratunga C, Khim N, Sreng S, Chim P, Kim S, et al Novel phenotypic assays for the detection of artemisinin-resistant Plasmodium falciparum malaria in Cambodia: in-vitro and ex-vivo drug-response studies. Lancet Infect Dis. 2013;13(12):1043–9. doi: 10.1016/S1473-3099(13)70252-4 . 24035558 10.1016/S1473-3099(13)70252-4 PMC5015432

[25] Meshnick S. Perspective: artemisinin-resistant malaria and the wolf. Am J Trop Med Hyg. 2012;87(5):783–4. doi: 10.4269/ajtmh.2012.12-0388 ; PubMed Central PMCID: PMC3516250. 23136171 10.4269/ajtmh.2012.12-0388 PMC3516250

[26] Roberts L. Drug-resistant malaria advances in Mekong. Science. 2017;358(6360):155–6. Epub 2017/10/14. doi: 10.1126/science.358.6360.155 . 29026020 10.1126/science.358.6360.155

[27] Krishna S, Kremsner PG. Antidogmatic approaches to artemisinin resistance: reappraisal as treatment failure with artemisinin combination therapy. Trends Parasitol. 2013;29(7):313–7. Epub 2013/04/30. doi: 10.1016/j.pt.2013.04.001 . 23623760 10.1016/j.pt.2013.04.001

[28] Dondorp AM, Ringwald P. Artemisinin resistance is a clear and present danger. Trends Parasitol. 2013;29(8):359–60. Epub 2013/06/19. doi: 10.1016/j.pt.2013.05.005 . 23768981 10.1016/j.pt.2013.05.005

[29] Saunders DL, Vanachayangkul P, Lon C, Program USAMMR, National Center for Parasitology E, Malaria C, et al Dihydroartemisinin-piperaquine failure in Cambodia. N Engl J Med. 2014;371(5):484–5. Epub 2014/07/31. doi: 10.1056/NEJMc1403007 . 25075853 10.1056/NEJMc1403007

[30] Nosten F, White NJ. Artemisinin-based combination treatment of falciparum malaria. Am J Trop Med Hyg. 2007;77(6 Suppl):181–92. Epub 2008/01/31. . 18165491

[31] Amaratunga C, Lim P, Suon S, Sreng S, Mao S, Sopha C, et al Dihydroartemisinin-piperaquine resistance in Plasmodium falciparum malaria in Cambodia: a multisite prospective cohort study. Lancet Infect Dis. 2016;16(3):357–65. doi: 10.1016/S1473-3099(15)00487-9 ; PubMed Central PMCID: PMCPMC4792715. 26774243 10.1016/S1473-3099(15)00487-9 PMC4792715

[32] Chaorattanakawee S, Lon C, Jongsakul K, Gawee J, Sok S, Sundrakes S, et al Ex vivo piperaquine resistance developed rapidly in Plasmodium falciparum isolates in northern Cambodia compared to Thailand. Malar J. 2016;15(1):519 Epub 2016/10/23. doi: 10.1186/s12936-016-1569-y ; PubMed Central PMCID: PMCPMC5075182. 27769299 10.1186/s12936-016-1569-y PMC5075182

[33] Witkowski B, Duru V, Khim N, Ross LS, Saintpierre B, Beghain J, et al A surrogate marker of piperaquine-resistant Plasmodium falciparum malaria: a phenotype-genotype association study. Lancet Infect Dis. 2017;17(2):174–83. Epub 2016/11/08. doi: 10.1016/S1473-3099(16)30415-7 ; PubMed Central PMCID: PMCPMC5266792. 27818097 10.1016/S1473-3099(16)30415-7 PMC5266792

[34] Amato R, Lim P, Miotto O, Amaratunga C, Dek D, Pearson RD, et al Genetic markers associated with dihydroartemisinin-piperaquine failure in Plasmodium falciparum malaria in Cambodia: a genotype-phenotype association study. Lancet Infect Dis. 2017;17(2):164–73. Epub 2016/11/08. doi: 10.1016/S1473-3099(16)30409-1 . 27818095 10.1016/S1473-3099(16)30409-1 PMC5564489

[35] Amato R, Pearson RD, Almagro-Garcia J, Amaratunga C, Lim P, Suon S, et al Origins of the current outbreak of multidrug resistant malaria in Southeast Asia: a retrospective genetic study. bioRxiv. 2017 doi: 10.1101/208371 10.1016/S1473-3099(18)30068-9 PMC5835763 29398391

[36] Antimalarial drug resistance in the Greater Mekong Subregion: How concerned should we be? [Internet]. 2017. World Health Organization; 29 September 2017. Available from: http://www.who.int/malaria/media/drug-resistance-greater-mekong-qa/en/

[37] Efficacy, Safety, and Tolerability of Dihydroartemisinin-piperaquine + Mefloquine Compared to Dihydroartemisinin-piperaquine or Artesunate-mefloquine in Patients With Uncomplicated Falciparum Malaria in Cambodia [Internet]. [cited November 26, 2017]. Available from: https://clinicaltrials.gov/ct2/show/NCT02612545.

[38] Study of Artemether-lumefantrine, Amodiaquine and Primaquine in Healthy Subjects (ALAQPQ) [Internet]. [cited November 26, 2017]. Available from: https://clinicaltrials.gov/ct2/show/NCT02696954.

[39] O'Neill PM, Rawe SL, Borstnik K, Miller A, Ward SA, Bray PG, et al Enantiomeric 1,2,4-trioxanes display equivalent in vitro antimalarial activity versus Plasmodium falciparum malaria parasites: implications for the molecular mechanism of action of the artemisinins. Chembiochem. 2005;6(11):2048–54. Epub 2005/10/14. doi: 10.1002/cbic.200500048 . 16222725 10.1002/cbic.200500048

[40] Charman SA, Arbe-Barnes S, Bathurst IC, Brun R, Campbell M, Charman WN, et al Synthetic ozonide drug candidate OZ439 offers new hope for a single-dose cure of uncomplicated malaria. Proc Natl Acad Sci U S A. 2011;108(11):4400–5. Epub 2011/02/09. doi: 10.1073/pnas.1015762108 ; PubMed Central PMCID: PMCPMC3060245. 21300861 10.1073/pnas.1015762108 PMC3060245

[41] Baumgartner F, Jourdan J, Scheurer C, Blasco B, Campo B, Maser P, et al In vitro activity of anti-malarial ozonides against an artemisinin-resistant isolate. Malar J. 2017;16(1):45 Epub 2017/01/27. doi: 10.1186/s12936-017-1696-0 ; PubMed Central PMCID: PMCPMC5267415. 28122617 10.1186/s12936-017-1696-0 PMC5267415

[42] Jimenez-Diaz MB, Ebert D, Salinas Y, Pradhan A, Lehane AM, Myrand-Lapierre ME, et al (+)-SJ733, a clinical candidate for malaria that acts through ATP4 to induce rapid host-mediated clearance of Plasmodium. Proc Natl Acad Sci U S A. 2014;111(50):E5455–62. Epub 2014/12/03. doi: 10.1073/pnas.1414221111 ; PubMed Central PMCID: PMCPMC4273362. 25453091 10.1073/pnas.1414221111 PMC4273362

[43] Mbengue A, Bhattacharjee S, Pandharkar T, Liu H, Estiu G, Stahelin RV, et al A molecular mechanism of artemisinin resistance in Plasmodium falciparum malaria. Nature. 2015;520(7549):683–7. doi: 10.1038/nature14412 ; PubMed Central PMCID: PMC4417027. 25874676 10.1038/nature14412 PMC4417027

[44] Mok S, Ashley EA, Ferreira PE, Zhu L, Lin Z, Yeo T, et al Drug resistance. Population transcriptomics of human malaria parasites reveals the mechanism of artemisinin resistance. Science. 2015;347(6220):431–5. doi: 10.1126/science.1260403 . 25502316 10.1126/science.1260403 PMC5642863

[45] Dogovski C, Xie SC, Burgio G, Bridgford J, Mok S, McCaw JM, et al Targeting the cell stress response of Plasmodium falciparum to overcome artemisinin resistance. PLoS Biol. 2015;13(4):e1002132 doi: 10.1371/journal.pbio.1002132 ; PubMed Central PMCID: PMC4406523. 25901609 10.1371/journal.pbio.1002132 PMC4406523

[46] Li H, O'Donoghue AJ, van der Linden WA, Xie SC, Yoo E, Foe IT, et al Structure- and function-based design of Plasmodium-selective proteasome inhibitors. Nature. 2016;530(7589):233–6. Epub 2016/02/13. doi: 10.1038/nature16936 ; PubMed Central PMCID: PMCPMC4755332. 26863983 10.1038/nature16936 PMC4755332

[47] Ismail HM, Barton V, Phanchana M, Charoensutthivarakul S, Wong MH, Hemingway J, et al Artemisinin activity-based probes identify multiple molecular targets within the asexual stage of the malaria parasites Plasmodium falciparum 3D7. Proc Natl Acad Sci U S A. 2016;113(8):2080–5. doi: 10.1073/pnas.1600459113 ; PubMed Central PMCID: PMCPMC4776496. 26858419 10.1073/pnas.1600459113 PMC4776496

[48] Wang J, Zhang CJ, Chia WN, Loh CC, Li Z, Lee YM, et al Haem-activated promiscuous targeting of artemisinin in Plasmodium falciparum. Nat Commun. 2015;6:10111 doi: 10.1038/ncomms10111 ; PubMed Central PMCID: PMCPMC4703832. 26694030 10.1038/ncomms10111 PMC4703832

[49] Ecker A, Lehane AM, Clain J, Fidock DA. PfCRT and its role in antimalarial drug resistance. Trends Parasitol. 2012;28(11):504–14. Epub 2012/10/02. doi: 10.1016/j.pt.2012.08.002 ; PubMed Central PMCID: PMCPMC3478492. 23020971 10.1016/j.pt.2012.08.002 PMC3478492

